# Lifecourse socioeconomic status and type 2 diabetes: the role of chronic inflammation in the English Longitudinal Study of Ageing

**DOI:** 10.1038/srep24780

**Published:** 2016-04-22

**Authors:** Silvia Stringhini, Paola Zaninotto, Meena Kumari, Mika Kivimäki, G. David Batty

**Affiliations:** 1 Institute of Social and Preventive Medicine (IUMSP), Lausanne University Hospital, Lausanne, Switzerland; 2 University College London, Department of Epidemiology and Public Health, London, United Kingdom; 3 Institute for Social and Economic Research, University of Essex, Essex, United Kingdom

## Abstract

We examined the association between lifecourse socioeconomic status (SES) and the risk of type 2 diabetes at older ages, ascertaining the extent to which adult lifestyle factors and systemic inflammation explain this relationship. Data were drawn from the English Longitudinal Study of Ageing (ELSA) which, established in 2002, is a representative cohort study of ≥50-year olds individuals living in England. SES indicators were paternal social class, participants’ education, participants’ wealth, and a lifecourse socioeconomic index. Inflammatory markers (C-reactive protein and fibrinogen) and lifestyle factors were measured repeatedly; diabetes incidence (new cases) was monitored over 7.5 years of follow-up. Of the 6218 individuals free from diabetes at baseline (44% women, mean aged 66 years), 423 developed diabetes during follow-up. Relative to the most advantaged people, those in the lowest lifecourse SES group experienced more than double the risk of diabetes (hazard ratio 2.59; 95% Confidence Interval (CI) = 1.81–3.71). Lifestyle factors explained 52% (95%CI:30–85) and inflammatory markers 22% (95%CI:13–37) of this gradient. Similar results were apparent with the separate SES indicators. In a general population sample, socioeconomic inequalities in the risk of type 2 diabetes extend to older ages and appear to partially originate from socioeconomic variations in modifiable factors which include lifestyle and inflammation.

Based on Global Burden of Disease (GBD) estimates, type 2 diabetes (hereafter referred to as ‘diabetes’) has led to more than 12 million deaths in 2010, a 93% rise from 1990^1^. Diabetes is also a major cause of disability, with an estimated 46,823 disability-adjusted years of life lost (DALYs) in 2010 (a 69% increase from 1990)^2^. While low socioeconomic status (SES) across the lifecourse has been found to be a major risk factor for the development of diabetes in adult life^3^,^4^,^5^,^6^,^7^,^8^,^9^, the mechanisms underlying this relationship are not fully understood^4^. Socioeconomic adversity in early life has been related to increased inflammatory activity in adult life^10^,^11^,^12^,^13^,^14^,^15^ and inflammation itself has been found to be associated with future diabetes risk^16^,^17^,^18^. Inflammation may also increase diabetes risk indirectly via other factors, including obesity^19^. Chronic inflammation may therefore be a mediator of the association between socioeconomic adversity and diabetes. Indeed, in the Whitehall II study of employed British middle-aged adults, chronic inflammation partially explained the association between lifecourse socioeconomic status and diabetes incidence^5^. However, it remains to be established whether the same factors explain socioeconomic differences in diabetes at older ages and in the general population which represents the full range of socioeconomic variation. The English Longitudinal Study of Ageing (ELSA) provides an opportunity to answer these questions. ELSA is also unusual in having repeated measurement of lifestyle risk factors for diabetes, such as obesity and physical activity, and inflammatory markers, allowing for more accurate characterisation of these potential explanatory variables than has previously been possible.

## Data and Methods

Data are drawn from ELSA, a prospective cohort study of community-dwelling older people. Described in detail elsewhere^20^, ELSA was established in 2002 with a core sample of 11,391 women and men aged 50 years and over, and was found to be representative of the national population in this age band living in private addresses in England^20^. Participants are contacted every 2 years for a follow-up interview and every 4 years for a medical examination. For the present analysis, ‘baseline’ was fixed at the ELSA wave 2 (2004/5) when biological data were first collected. ELSA was approved by the London Multicentre Research Ethics Committee (MREC/01/2/91) and informed consent was obtained from all participants. The methods were carried out in accordance with the approved guidelines.

Four indicators of socioeconomic status (SES) were used. Both paternal social class (high [managerial, professional, and administrative occupations or business owners], intermediate [trade- and services related occupations], and low [manual and casual occupations and other occupations]), and education (high [finished full-time education at ≥17 years], intermediate [finished full-time education at 15–16 years], and low [finished full-time education at ≤14 years or no education]) were divided into three levels. Our third SES variable was total net non-pension household wealth which was categorised into tertiles and based on an estimation of the assets of study members and their partners, including properties, businesses, other assets, and any form of investments or savings (except for pension savings) less debts owed by them. The fourth SES indicator included was a composite lifecourse SES variable. To compute this, paternal social class, education and adult wealth were coded as 0–2 with higher values indicating greater disadvantage. The three SES indicators were then summed, resulting in a 7-level score with higher values corresponding to higher lifecourse disadvantage.

At study waves 2 (2004/5), 4 (2008/9) and 6 (2012/13) venous blood was taken by a research nurse. Using these data, diabetes was defined as blood glycated haemoglobin level (HbA1C) ≥48 mmol/mol (6.5%), following the World Health Organization (WHO) recommendations^21^. For participants with missing values on HbA1C, we used self-reported doctor-diagnosed diabetes, as we have previously^4^,^8^. The date of diabetes diagnosis was assigned according to the interval method, whereby the midpoint between the first visit with a diabetes diagnosis and the last visit without diabetes was used^22^.

C-reactive protein (CRP) was analyzed by using the N Latex CRP mono Immunoassay on the Behring Nephelometer II Analyzer (Dade Behring, Milton Keynes, UK). In all analyses, CRP data, originally skewed, were normalized using log-transformation. Participants with CRP levels >10 mg/l were excluded from the analysis as they may indicate current infection instead of chronic inflammation. Fibrinogen was analyzed using a modification of the Clauss thrombin clotting method on the Organon Teknika MDA 180 analyzer. In all analyses, CRP and fibrinogen were treated as continuous variables. Current smoking was self-reported and classified as current, former, never smoker. Physical activity was assessed by asking participants how often they engaged in vigorous, moderate or mild physical activity. Three groups were the created: active (1/week), moderately active (1–3 times/month), and inactive (hardly ever/never). Alcohol intake was self-reported and classified <daily or daily. Height and weight were measured directly using standard procedures; Body Mass Index (BMI) was then computed and three classifications produced (<25; 25–29; ≥30 kg/m^2^)^23^. Ethnicity (white/non-white), based on wave 2 data, as well as age and sex were treated as confounders in the analyses. All effect estimates were controlled for prevalent chronic illness (coronary heart disease, stroke and/or cancer) to account for the potential effect of these comorbidities.

### Statistical analysis

Missing values for lifestyle factors and inflammatory markers were imputed using information collected at the previous or successive wave. For those without information available at adjacent waves, multivariate imputation based on sex, age, ethnicity, lifestyle factors and inflammatory markers (Stata *uvis* procedure) was used. Missing values on SES indicators and diabetes, our exposure and outcome, respectively, were not imputed. The association between SES and diabetes risk factors at baseline was examined using age-, sex-, ethnicity and prevalent conditions adjusted logistic regressions for dichotomous variables (smoking, physical inactivity, alcohol consumption) and linear regressions for continuous variables (BMI, fibrinogen and CRP). Having ascertained that the proportional hazard assumption had not been violated, Cox regressions were used to compute hazard ratio (HR) with accompanying 95% confidence intervals (CI) for the association between lifecourse SES and diabetes. The association between SES and diabetes was first examined with a model including adjustment for age, ethnicity and an interaction term which was used to ascertain if sex modified the SES-diabetes relationship. As the effect of SES on type 2 diabetes did not differ by sex (p-value for interaction = 0.586), data were pooled and our effect estimates sex-adjusted (supplementary information). As tests did not suggest departure from a linear trend (p ≥0.05), SES indicators were entered into the models as continuous variables. The hazard ratio (HR) associated with a unit change in SES was modified in order to yield the HR in the lowest versus the highest SES category for each SES indicator. Each SES indicator was first entered in a basic model containing age, sex, ethnicity, and prevalent conditions (Model 1). Lifestyle factors and inflammatory markers (time-dependent covariates updated at waves 2 and 4) were then entered individually and simultaneously into Model 1. Further, to account for long-term exposure to these risk factors, at each follow-up period we controlled not only for the risk factor at the current wave but also for the risk factors at previous waves, as we have previously^5^. The contribution of risk factors in explaining the SES- diabetes association was determined by the percent attenuation in the β coefficient for SES after inclusion of the risk factor in question to Model 1: “100 × (β_Model 1_ − β_Model 1+risk factor(s)_)/(β_Model 1_)”. The independent contribution of inflammatory markers (from lifestyle factors) is calculated as “100 × (β_Model 1+lifestyle factors_ − β_Model 1+all factors_)/(β_Model 1+lifestyle factors_)”. We calculated a 95% confidence interval (CI) around the percentage attenuation using a bootstrap method with 1000 re-samplings.

## Results

A total of 8688 participants attended ELSA wave 2 (study baseline). Of these, 2470 were excluded because of missing values (missingness was not mutually exclusive) on socioeconomic indicators (N = 89), behavioural factors or inflammatory markers (N = 1028), unknown diabetes status (N = 10), prevalent diabetes at baseline (N = 800) or CRP levels ≥10 mg/l (N = 1626). Excluded participants were older, more likely to be women, and had a lower SES (p < 0.05), although absolute differences were small.

In Table 1 we summarise the baseline characteristics of the 6218 participants included in the present analysis, 423 of whom developed diabetes over the 7.5 years mean follow-up (diabetes incidence rate 9.0 per 1000 person-years). Mean age at baseline was 65.8 years, 44% of participants were women, and the large majority was white European. A total of 14.2% of study members reported chronic disease conditions, the prevalence being highest in the lower SES groups (p < 0.05). Diabetes incidence was inversely associated with SES, such that highest rates were apparent among the disadvantaged as indexed by paternal social class, adult wealth, education, and lifecourse SES. With the exception of education, these effects were incremental across the full socioeconomic range.

The association between lifecourse socioeconomic indicators and diabetes risk factors at baseline is presented in Table 2. For the four SES indicators, individuals with a lowest vs highest SES were more likely to be smokers (OR = 8.90, 95%CI:6.60;12.0 for lifecourse SES score) and physically inactive (OR = 5.67, 95%CI:4.21;7.67 for lifecourse SES score), but less likely to consume alcohol frequently (OR = 0.17, 95%CI:0.13;0.21 for lifecourse SES score). They also had a higher BMI (β = 2.16, 95%CI:1.74;2.59 for lifecourse SES score), fibrinogen (β = 0.28, 95%CI:0.22;0.33 for lifecourse SES score) and CRP levels (β = 0.57, 95%CI:0.49;0.63 for lifecourse SES score).

**Figure 1:**
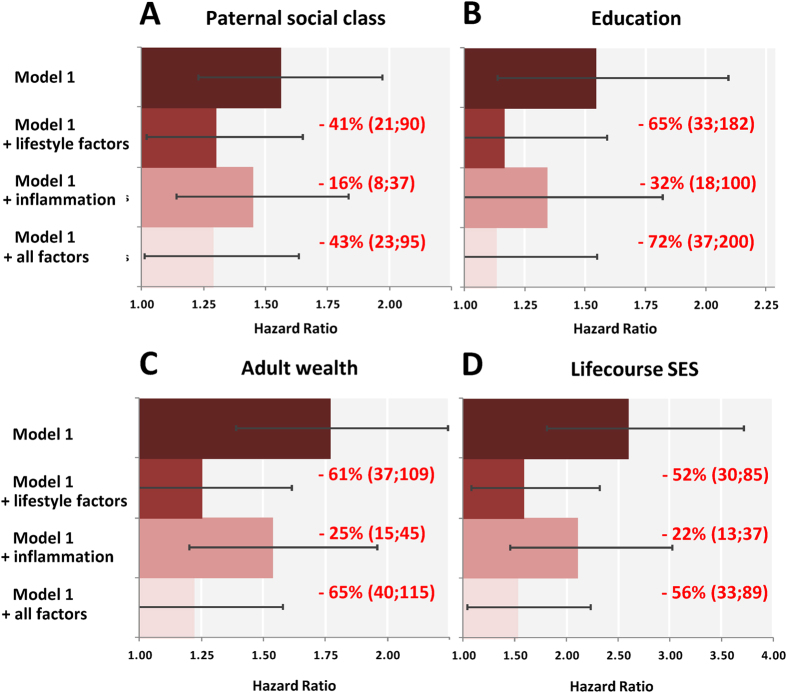
Hazard ratios (95% confidence intervals) for the relation of different indicators of SES with diabetes incidence. The English Longitudinal Study of Ageing 2004–2013. Hazard ratios are for the lowest vs. highest SES category. From top to bottom, the first bars shows hazard ratios for the relation of each SES indicator with diabetes, adjusted for age, sex, ethnicity and prevalent disease (model 1). The second bars show Model 1 with the addition of lifestyle factors (smoking, alcohol intake, physical activity, BMI); the third bars Model 1 with the addition of inflammatory markers (CRP and fibrinogen); and the fourth bars Model 1 with the combined addition of lifestyle factors and inflammatory markers. The contribution of inflammatory markers to the SES-diabetes gradient, independent from lifestyle factors, is 3% for paternal social class, 18% for education, 11% for wealth and 8% for lifecourse SES.

In Fig. 1 we show the age-, sex-, ethnicity- and prevalent health conditions-adjusted associations of SES indicators with diabetes incidence, together with the relative contribution of lifestyle factors and inflammatory markers to these gradients. Individuals in the most disadvantaged SES groups experienced a higher risk of developing diabetes during follow-up compared to those with the most advantaged groups for paternal social class (HR; 95% CI:1.56; 1.23–1.97), education (1.76; 1.39–2.24) and wealth (2.59; 1.82–3.71). Adding lifestyle factors to the multivariable models resulted in marked attenuation ranging from 41–65% depending on the SES indicator, whereas the explanatory power of inflammatory markers was more modest (16–32%). The additional contribution of inflammatory markers to the model already adjusted for lifestyle factors was 3% for paternal social class, 18% for education, 11% for wealth and 8% for lifecourse SES.

## Discussion

In this representative sample of the older English population, various indicators of low socioeconomic status across the lifecourse were related to an increased risk of later diabetes. Chronic inflammation explained up to one third of this association, while lifestyle factors explained up to two thirds, suggesting that much of these differences are modifiable. To our knowledge, this is the first examination of the explanatory power of systemic inflammation in the SES-diabetes relation in a population-representative sample of older ages. A further unusual feature of the present study was the repeat assessment of lifestyle and inflammatory markers during follow-up, thus reducing measurement error and accounting for potential changes in time-varying characteristics.

Our findings have important implications as recent studies suggest that gene regulation of the immune function may be one of the pathways through which socioeconomic status across the lifecourse is biologically embedded^10^,^24^. There are at least two biological explanations for the apparent mediating role of inflammation in the lifecourse SES- diabetes association. First, SES could affect inflammation through stress-mediated factors involving the hypothalamic-pituitary-adrenal axis and the autonomic nervous system^11^,^12^. Recent evidence reporting SES-related epigenetic changes in genomic regions regulating response to stress supports this possibility^24^,^25^,^26^. Second, inflammatory processes are related to several risk factors for diabetes that are strongly patterned by SES, such as obesity, unhealthy diet, and physical inactivity^27^,^28^,^29^. In addition, chronic stress in people experiencing socioeconomic adversity may influence lifestyle factors and inflammatory activity^30^. Finally, whether socioeconomic differences in inflammation arise late in life as a consequence of unhealthy behaviors and chronic stress, or are set in early life through an epigenetic programming of a defensive phenotype driven by adverse early life conditions, remains a matter of debate. Our findings of an independent contribution of inflammation (from lifestyle factors) to social inequalities in diabetes incidence and the stronger associations observed for the lifecourse SES indicator compared to childhood or adulthood SES indicators suggest that both mechanisms may be at play. Studies which capture additional inflammatory markers, such as interleukins and TNF-α, are now needed in order to obtain a more precise estimation of their role in social inequalities in type 2 diabetes and other inflammation-related diseases.

## Additional Information

**How to cite this article**: Stringhini, S. *et al*. Lifecourse socioeconomic status and type 2 diabetes: the role of chronic inflammation in the English Longitudinal Study of Ageing. *Sci. Rep.* **6**, 24780; doi: 10.1038/srep24780 (2016).

## Supplementary Material

Supplementary Information

## Figures and Tables

**Table 1:** Study participant characteristics at baseline and diabetes incidence at 7.5-year follow-up according to indicators of socioeconomic status in early and adult life.

	**N (%)**	**Age Mean (SD)**	**Men N (%)**	**White N (%)**	**Existing illness N (%)**	**Diabetes incidence N (rate**^b^)
Paternal social class						
High	1951 (31.4)	65.4 (8.7)	1147 (58.8)	1901 (97.4)	268 (13.7)	109 (6.4)
Middle	2009 (32.3)	65.9 (9.4)	1098 (54.7)	1994 (99.3)	271 (13.5)	128 (9.8)
Low	2258 (36.3)	66.2 (10.3)	1240 (54.9)	2221 (98.4)	343 (15.2)	186 (12.2)
p-value^a^		0.010	0.013	<0.001	0.224	0.002
Education						
High	1848 (29.7)	63.9 (9.2)	1031 (55.8)	1786 (96.7)	204 (11.0)	96 (6.6)
Middle	3250 (52.3)	63.6 (7.9)	1839 (56.6)	3222 (99.1)	418 (12.9)	249 (10.5)
Low	1120 (18.0)	75.7 (7.8)	615 (54.9)	1108 (98.9)	260 (23.2)	78 (9.7)
p-value		<0.001	0.681	<0.001	<0.001	0.076
Adult wealth						
High	2298 (37.0)	64.7 (8.8)	1220 (53.1)	2266 (98.6)	267 (11.6)	148 (6.6)
Middle	2136 (34.3)	65.7 (9.4)	1191 (55.8)	2114 (98.9)	306 (14.3)	98 (10.2)
Low	1783 (28.7)	67.5 (10.2)	1073 (60.2)	1735 (97.3)	309 (17.3)	177 (12.1)
p-value		<0.001	<0.001	<0.001	<0.001	0.001
Lifecourse SES						
0 (Highest)	668 (10.7)	63.6 (8.5)	378 (56.6)	651 (97.5)	67 (10.0)	27 (5.3)
1	845 (13.6)	64.3 (9.0)	466 (55.2)	830 (98.2)	100 (11.8)	40 (5.7)
2	1065 (17.1)	64.7 (9.0)	567 (53.2)	1043 (97.9)	134 (12.6)	75 (9.2)
3	1263 (20.3)	64.9 (8.9)	740 (58.6)	1249 (98.9)	165 (13.1)	79 (8.4)
4	1215 (19.5)	66.0 (9.3)	664 (54.7)	1198 (98.6)	194 (15.9)	98 (11.0)
5	876 (14.1)	68.3 (10.0)	509 (58.1)	864 (98.6)	152 (17.3)	78 (14.2)
6 (Lowest)	285 (4.6)	76.3 (8.2)	160 (56.1)	280 (98.3)	70 (24.6)	26 (14.8)
p-value		<0.001	0.113	0.257	<0.001	0.001
Overall	6218	65.8 (9.5)	3485 (56.1)	6116 (98.4)	882 (14.2)	423 (9.0)

The English Longitudinal Study of Ageing (2004–2013). CI, 95% confidence interval; SD, standard deviation.

^a^*p-value* for linear trend across socioeconomic categories.

^b^Age- and sex-adjusted incidence rate per 1000 person-years over a 7.5 years mean follow-up.

**Table 2:** Association between lifecourse socioeconomic indicators and diabetes risk factors at baseline.

	**Lowest vs highest paternal social class**	**Lowest vs highest education**	**Lowest vs highest adult wealth**	**Lowest vs highest lifecourse SES**
	**OR**^a^ **(95% CI)**	**OR**^a^ **(95% CI)**	**OR**^a^ **(95% CI)**	**OR**^a^ **(95% CI)**
Current smoking (Ref.: never/former smoking)	1.84 (1.54; 2.21)	3.10 (2.40; 4.02)	4.87 (4.00; 5.91)	8.90 (6.60; 12.0)
Sedentary (Ref.: moderately active/active)	1.53 (1.27; 1.85)	2.49 (1.97; 3.15)	3.89 (3.16; 4.77)	5.67 (4.21; 7.67)
Frequent alcohol consumption (Ref.: less than daily)	0.53 (0.47; 0.61)	0.31 (0.26; 0.38)	0.33 (0.29; 0.38)	0.17 (0.13; 0.21)
	**β**^a^ **(95% CI)**	**β**^a^ **(95% CI)**	**β**^a^ **(95% CI)**	**β**^a^ **(95% CI)**
Body mass index	0.96 (0.69; 1.25)	1.13 (0.77; 1.49)	1.32 (1.03; 1.61)	2.16 (1.74; 2.59)
Fibrinogen	0.11 (0.08; 0.15)	0.14 (0.10; 0.19)	0.18 (0.15; 0.22)	0.28 (0.22; 0.33)
CRP	0.19 (0.14; 0.25)	0.35 (0.28; 0.42)	0.38 (0.32; 0.43)	0.57 (0.49; 0.63)

The English Longitudinal Study of Ageing (2004–2013). β: Beta coefficient; CI: Confidence Interval; CRP: C-reactive protein; OR: Odds Ratio; Ref: Reference.

^a^Effect estimates adjusted for age, sex, ethnicity and prevalent chronic disease.

## References

[b1] Lozano R. . Global and regional mortality from 235 causes of death for 20 age groups in 1990 and 2010: a systematic analysis for the Global Burden of Disease Study 2010. Lancet 380, 2095–2128, doi: 10.1016/S0140-6736(12)61728-0 (2012). 23245604 PMC10790329

[b2] Murray C. J. . Disability-adjusted life years (DALYs) for 291 diseases and injuries in 21 regions, 1990–2010: a systematic analysis for the Global Burden of Disease Study 2010. Lancet 380, 2197–2223, doi: 10.1016/S0140-6736(12)61689-4 (2012). 23245608

[b3] Agardh E., Allebeck P., Hallqvist J., Moradi T. & Sidorchuk A. Type 2 diabetes incidence and socio-economic position: a systematic review and meta-analysis. Int J Epidemiol 40, 804–818, doi: 10.1093/ije/dyr029 (2011). 21335614

[b4] Stringhini S. . Contribution of modifiable risk factors to social inequalities in type 2 diabetes: prospective Whitehall II cohort study. BMJ 345, e5452, doi: 10.1136/bmj.e5452 (2012). 22915665 PMC3424226

[b5] Stringhini S. . Association of Lifecourse Socioeconomic Status with Chronic Inflammation and Type 2 Diabetes Risk: The Whitehall II Prospective Cohort Study. PLoS Med 10, e1001479, doi: 10.1371/journal.pmed.1001479 (2013). 23843750 PMC3699448

[b6] Krishnan S., Cozier Y. C., Rosenberg L. & Palmer J. R. Socioeconomic status and incidence of type 2 diabetes: results from the Black Women’s Health Study. Am J Epidemiol 171, 564–570, doi: 10.1093/aje/kwp443 (2010). 20133518 PMC2842221

[b7] Smith B. T. . Life-course socioeconomic position and type 2 diabetes mellitus: The Framingham Offspring Study. Am J Epidemiol 173, 438–447, doi: 10.1093/aje/kwq379 (2011). 21242301 PMC3032804

[b8] Demakakos P., Marmot M. & Steptoe A. Socioeconomic position and the incidence of type 2 diabetes: the ELSA study. Eur J Epidemiol 27, 367–378, doi: 10.1007/s10654-012-9688-4 (2012). 22539241

[b9] Hsu C. C. . Poverty increases type 2 diabetes incidence and inequality of care despite universal health coverage. Diabetes Care 35, 2286–2292, doi: 10.2337/dc11-2052 (2012). 22912425 PMC3476930

[b10] Miller G. E. . Low early-life social class leaves a biological residue manifested by decreased glucocorticoid and increased proinflammatory signaling. Proc Natl Acad Sci USA 106, 14716–14721, doi: 10.1073/pnas.0902971106 (2009). 19617551 PMC2732821

[b11] Hemingway H. . Social and psychosocial influences on inflammatory markers and vascular function in civil servants (the Whitehall II study). Am J Cardiol 92, 984–987 (2003). 14556880 10.1016/s0002-9149(03)00985-8

[b12] Jousilahti P., Salomaa V., Rasi V., Vahtera E. & Palosuo T. Association of markers of systemic inflammation, C reactive protein, serum amyloid A, and fibrinogen, with socioeconomic status. J Epidemiol Community Health 57, 730–733 (2003). 12933781 10.1136/jech.57.9.730 PMC1732590

[b13] Alley D. E. . Socioeconomic status and C-reactive protein levels in the US population: NHANES IV. Brain Behav Immun 20, 498–504, doi: 10.1016/j.bbi.2005.10.003 (2006). 16330181

[b14] Loucks E. B. . Association of educational level with inflammatory markers in the Framingham Offspring Study. Am J Epidemiol 163, 622–628, doi: 10.1093/aje/kwj076 (2006). 16421236

[b15] Fraga S. . Association of socioeconomic status with inflammatory markers: a two cohort comparison. Prev Med 71, 12–19, doi: 10.1016/j.ypmed.2014.11.031 (2015). 25482420

[b16] Pradhan A. D., Manson J. E., Rifai N., Buring J. E. & Ridker P. M. C-reactive protein, interleukin 6, and risk of developing type 2 diabetes mellitus. JAMA 286, 327–334 (2001). 11466099 10.1001/jama.286.3.327

[b17] Hotamisligil G. S. Inflammation and metabolic disorders. Nature 444, 860–867, doi: 10.1038/nature05485 (2006). 17167474

[b18] Donath M. Y. . Mechanisms of beta-cell death in type 2 diabetes. Diabetes 54 Suppl 2, S108–113 (2005). 16306327 10.2337/diabetes.54.suppl_2.s108

[b19] Mohamed-Ali V. . Subcutaneous adipose tissue releases interleukin-6, but not tumor necrosis factor-alpha, *in vivo*. J Clin Endocrinol Metab 82, 4196–4200 (1997). 9398739 10.1210/jcem.82.12.4450

[b20] Steptoe A., Breeze E., Banks J. & Nazroo J. Cohort profile: the English longitudinal study of ageing. Int J Epidemiol 42, 1640–1648, doi: 10.1093/ije/dys168 (2013). 23143611 PMC3900867

[b21] World Health Organisation. Use of Glycated Haemoglobin (HbA1c) in the Diagnosis of Diabetes Mellitus. Abbreviated Report of a WHO Consultation (2011). 26158184

[b22] Tabak A. G. . Trajectories of glycaemia, insulin sensitivity, and insulin secretion before diagnosis of type 2 diabetes: an analysis from the Whitehall II study. Lancet 373, 2215–2221, doi: 10.1016/s0140-6736(09)60619-x (2009). 19515410 PMC2726723

[b23] World Health Organisation. The challenge of obesity in the WHO European Region and the strategies for response. 1 edn, (WHO, 2007).

[b24] Stringhini S. . Lifecourse socioeconomic status and DNA methylation of genes regulating inflammation. Int J Epidemiol in press (2015). 10.1093/ije/dyv060 25889032

[b25] Needham B. L. . Life course socioeconomic status and DNA methylation in genes related to stress reactivity and inflammation: The multi-ethnic study of atherosclerosis. Epigenetics 10, 958–969, doi: 10.1080/15592294.2015.1085139 (2015). 26295359 PMC4844216

[b26] Borghol N. . Associations with early-life socio-economic position in adult DNA methylation. Int J Epidemiol 41, 62–74, doi: 10.1093/ije/dyr147 (2012). 22422449 PMC3304522

[b27] Abramson J. L. & Vaccarino V. Relationship between physical activity and inflammation among apparently healthy middle-aged and older US adults. Arch Intern Med 162, 1286–1292 (2002). 12038947 10.1001/archinte.162.11.1286

[b28] Franks P. W. Obesity, inflammatory markers and cardiovascular disease: distinguishing causality from confounding. J Hum Hypertens 20, 837–840, doi: 10.1038/sj.jhh.1002059 (2006). 16855628

[b29] O’Connor M. F. . To assess, to control, to exclude: effects of biobehavioral factors on circulating inflammatory markers. Brain Behav Immun 23, 887–897, doi: 10.1016/j.bbi.2009.04.005 (2009). 19389469 PMC2749909

[b30] Scott K. A., Melhorn S. J. & Sakai R. R. Effects of Chronic Social Stress on Obesity. Current obesity reports 1, 16–25, doi: 10.1007/s13679-011-0006-3 (2012). 22943039 PMC3428710

