# Supplementary material for: Lifecourse socioeconomic status and type 2 diabetes: the role of chronic inflammation in the English Longitudinal Study of Ageing
Source: Sci Rep. 2016 Apr 22;6:24780. doi: 10.1038/srep24780 (PMC4840327; doi:10.1038/srep24780)
Supplement: Supplementary Information [file srep24780-s1.pdf]

Scientific Reports

Lifecourse socioeconomic status and type 2 diabetes: the role of chronic inflammation in the English Longitudinal Study of Ageing

Silvia Stringhini [1], Paola Zaninotto [2], Meena Kumari [3], Mika Kivimaki[2], G. David Batty[2]
[1] Institute of Social and Preventive Medicine (IUMSP), Lausanne University Hospital, Lausanne, Switzerland
[2] University College London, Department of Epidemiology and Public Health, London, United Kingdom
[3] Institute for Social and Economic Research, University of Essex, Essex

Running head: Socioeconomic status and type 2 diabetes at older ages

SUPPLEMENTARY FILES

Table S1. Association of lifecourse socioeconomic score with diabetes incidence ANALYSIS STRATIFED BY GENDER

	MEN		WOMEN	
	**HR (95%CI)**	**%Δ**	**HR (95%CI)**	**%Δ**
**Model 1:** Adjusted for age, sex, ethnicity, prevalent conditions	2.89 (1.76;4.75)		2.29 (1.36;3.87)	
**Model 2:** Model 1 + lifestyle factors	1.63 (0.96;2.76)	-54	1.51 (0.86;2.62)	-51
**Model 3:** Model 1 + inflammation	2.23 (1.34;3.69)	-25	1.97 (1.16; 3.36)	-18
**Model 4:** Model 1 + all factors	1.58 (0.93;2.68)	-57	1.45 (0.83; 2.55)	-55

Δ: Attenuation; BMI: Body Mass Index; CI: Confidence Interval; CRP: C-reactiv protein; HR: Hazard ratio
Lifestyle factors include smoking, alcohol intake, physical activity, BMI. Inflammation include fibrinogen and CRP.
